# Supplementary figures and images for: Brianolide from Briareum stechei Attenuates Atopic Dermatitis-like Skin Lesions by Regulating the NFκB and MAPK Pathways
Source: Biomolecules. 2025 Jun 14;15(6):871. doi: 10.3390/biom15060871 (PMC12190504; doi:10.3390/biom15060871)

Figure 5A

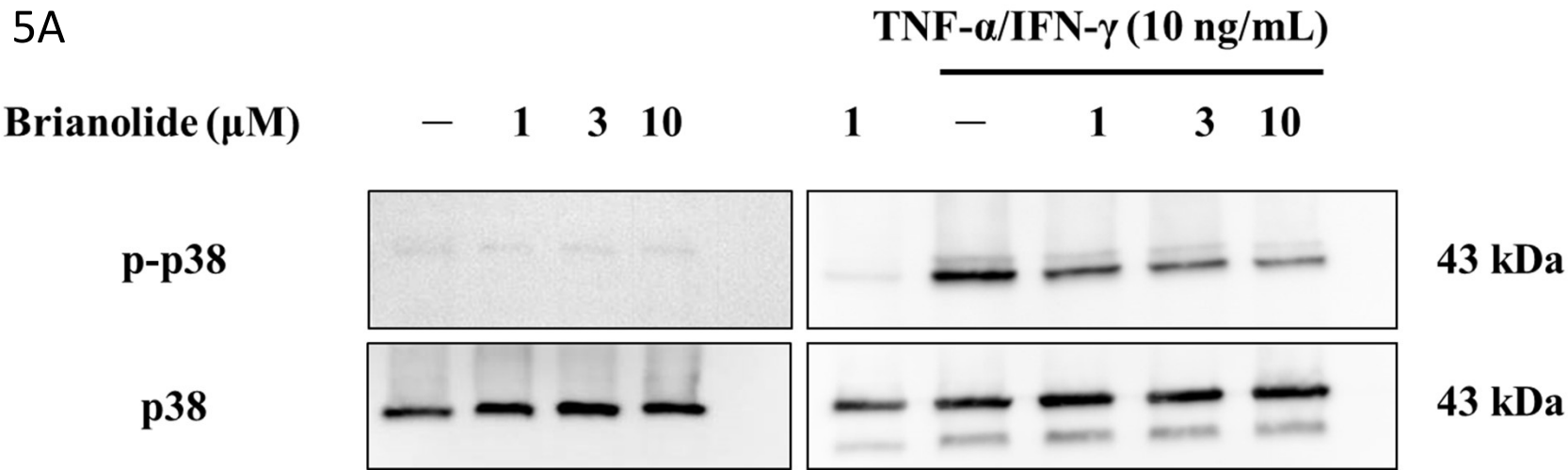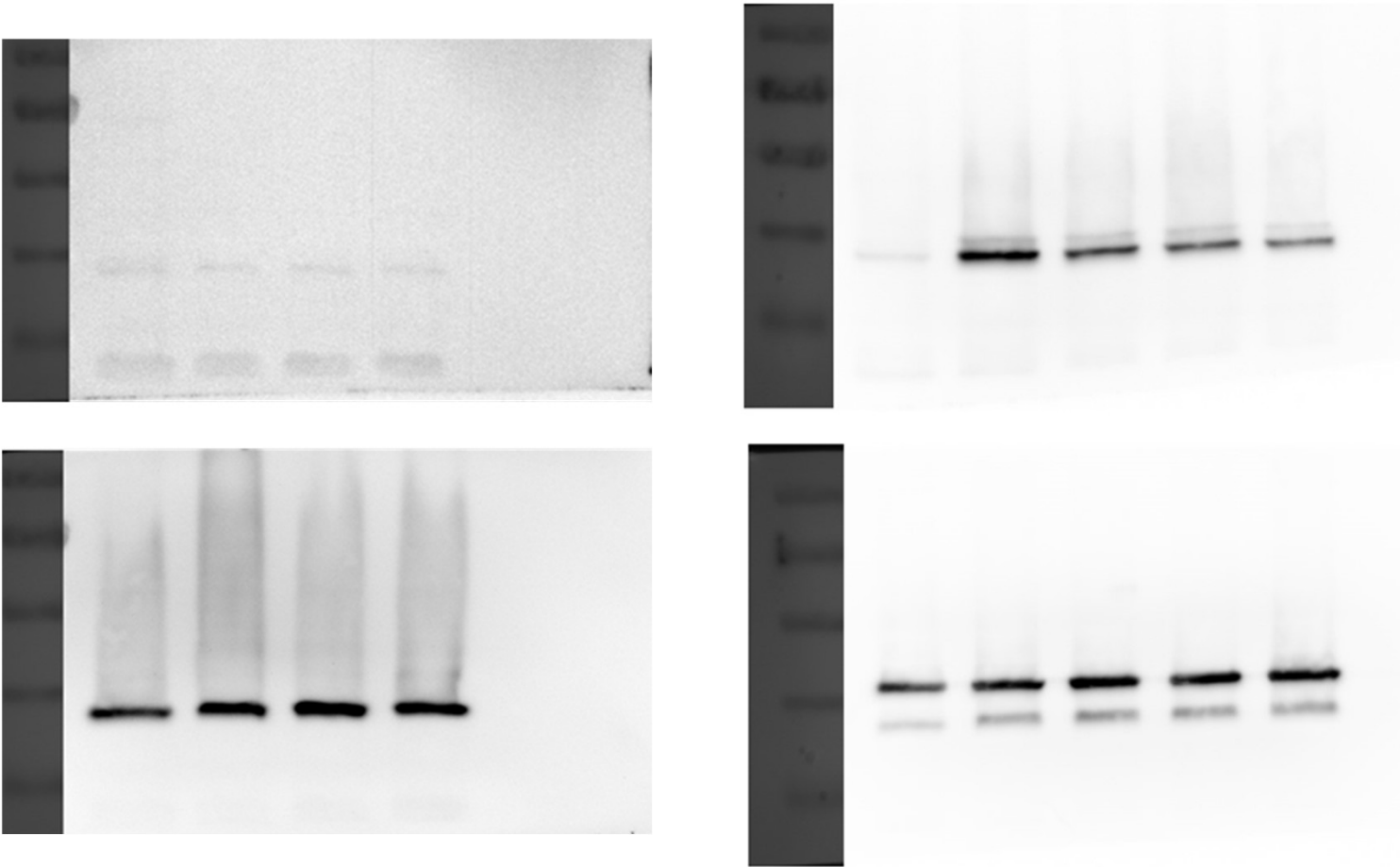

Figure 5B

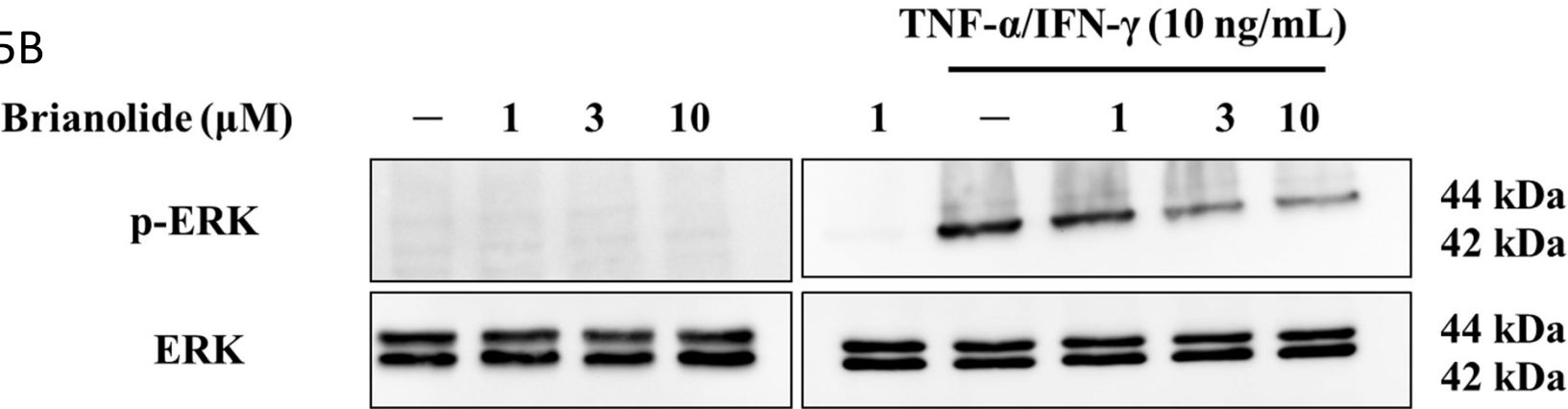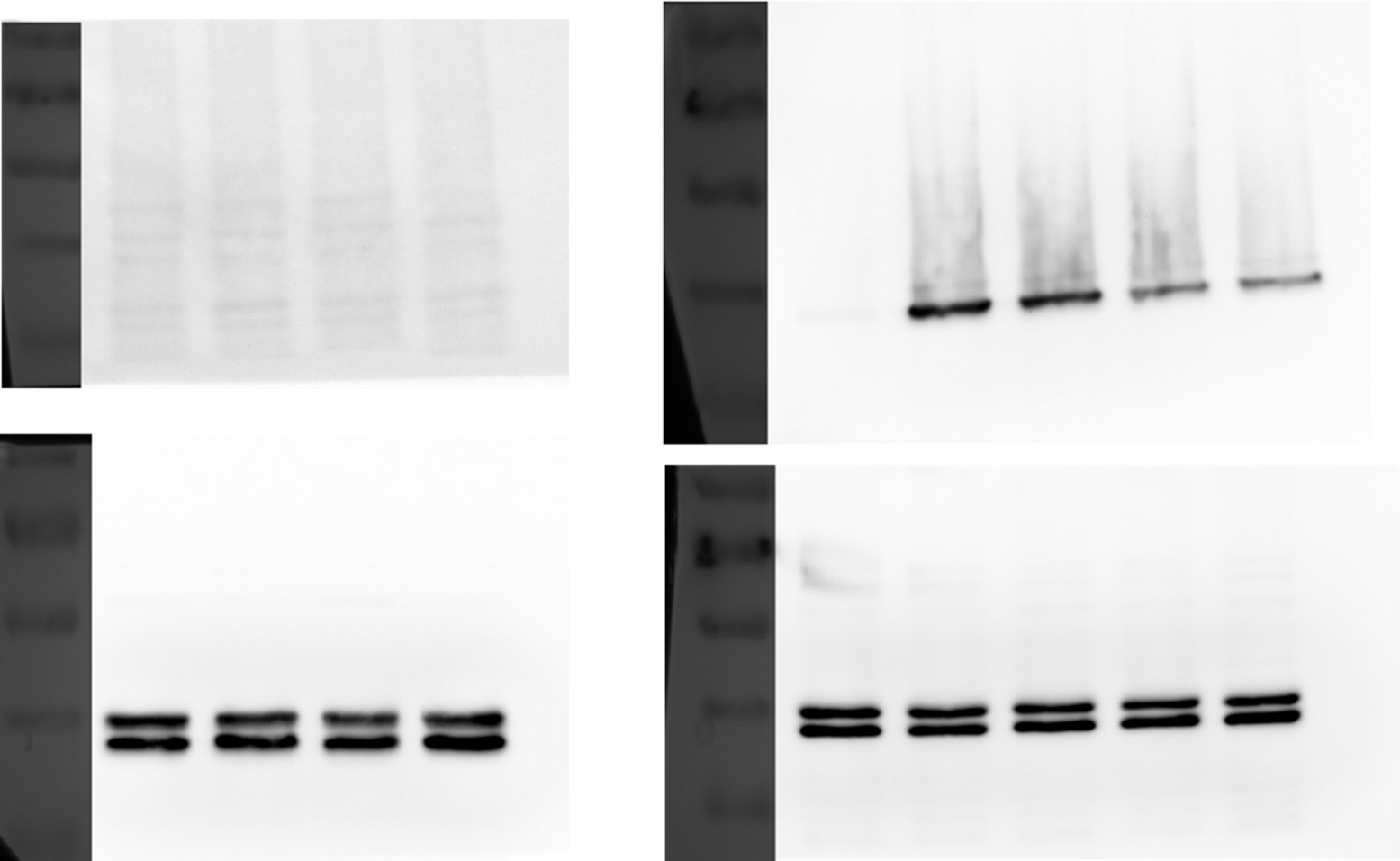

Figure 5C

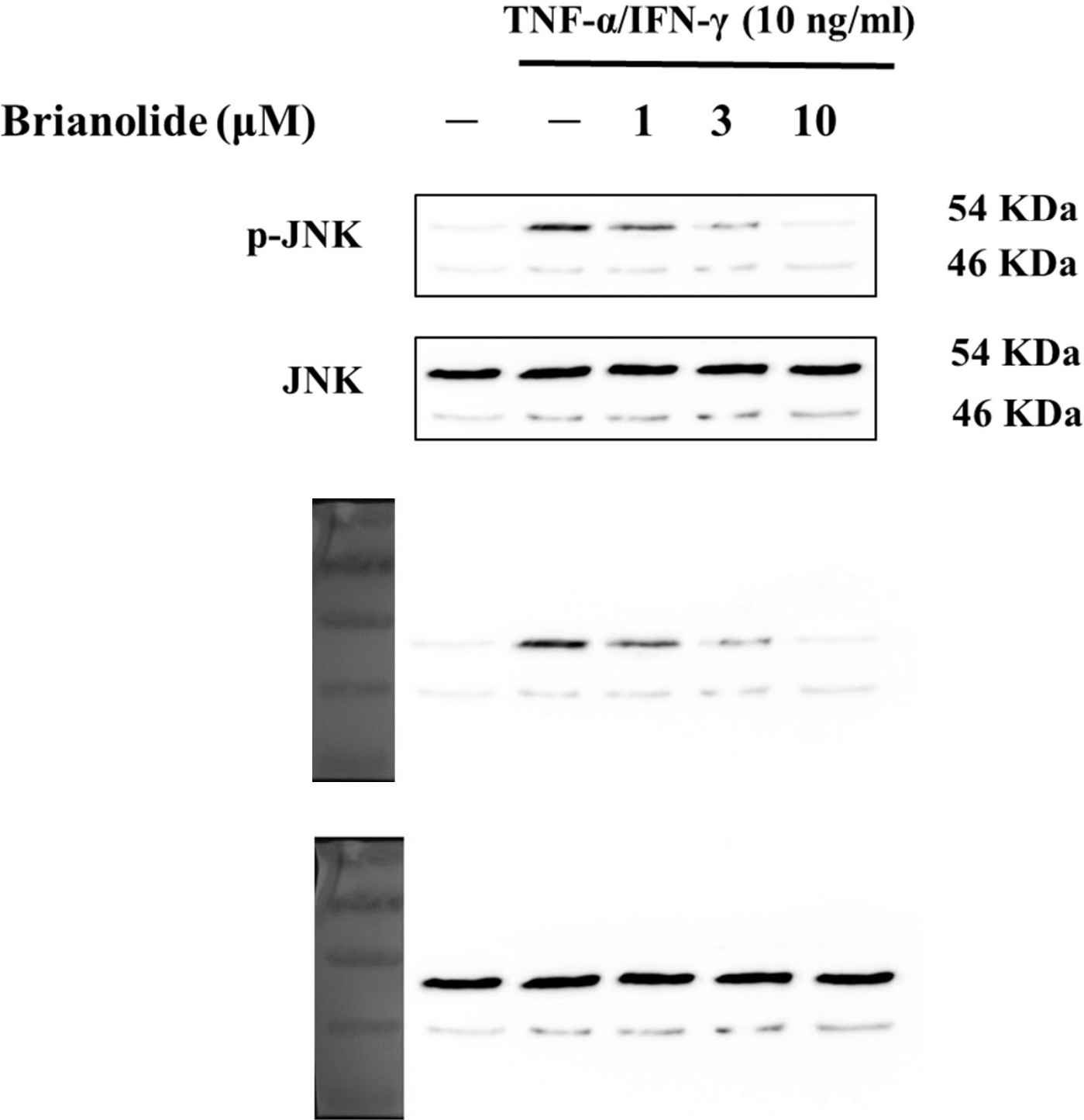

Figure 6A

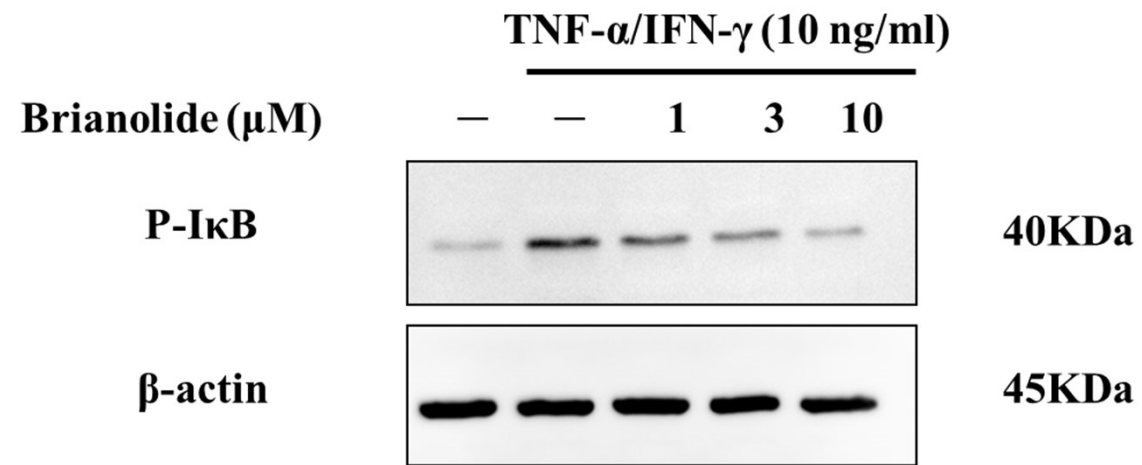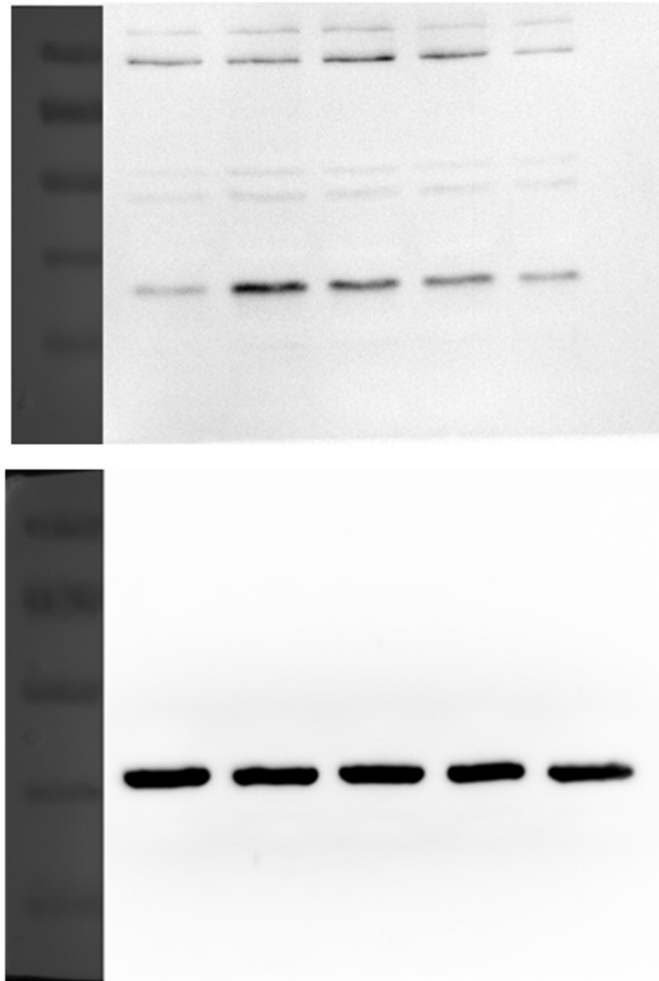

Figure 6B

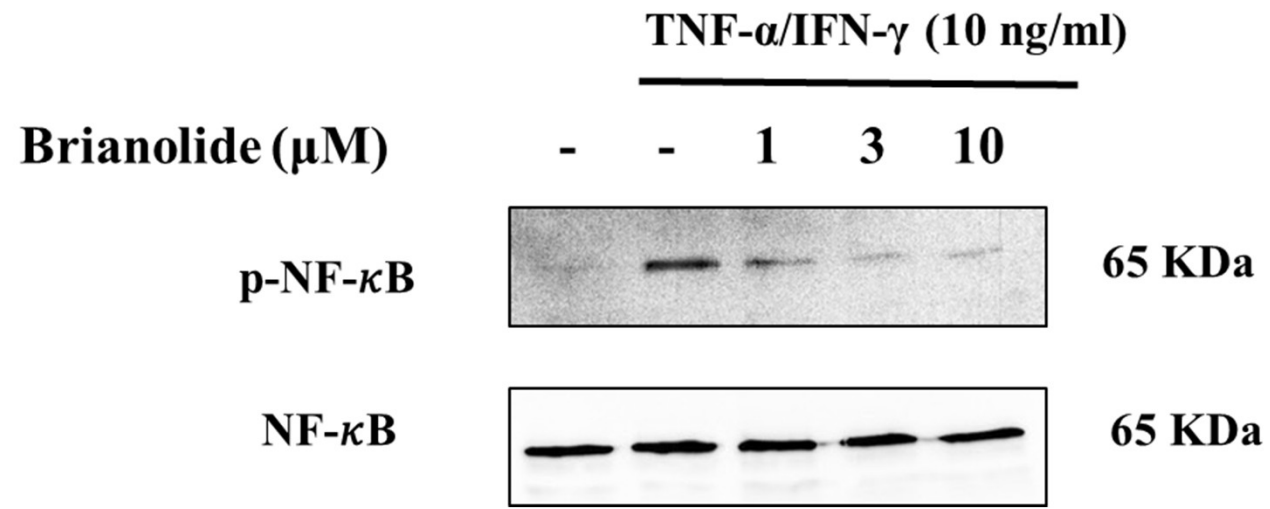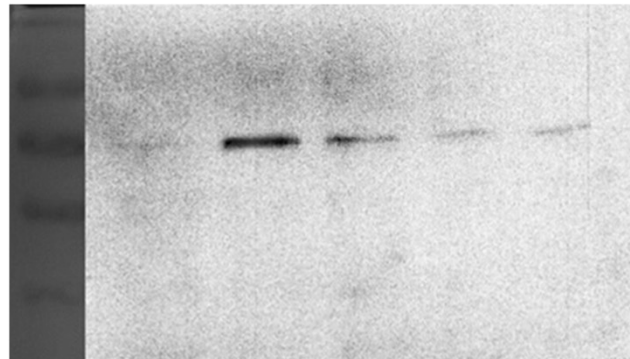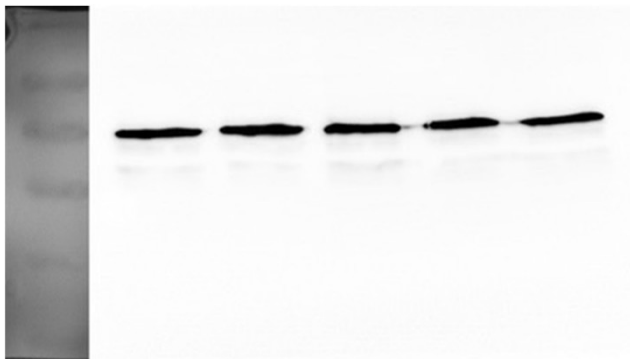

Supplement: Supplementary file 1 [file biomolecules-15-00871-s001.zip › biomolecules-3668161-supplementary-Western blot original images.pdf]
